# Poor sleep quality among newly diagnosed head and neck cancer patients: prevalence and associated factors

**DOI:** 10.1007/s00520-020-05577-9

**Published:** 2020-06-22

**Authors:** Angelina M. M. Santoso, Femke Jansen, Birgit I. Lissenberg-Witte, Robert J. Baatenburg de Jong, Johannes A. Langendijk, C. René Leemans, Johannes H. Smit, Robert P. Takes, Chris H. J. Terhaard, Annemieke van Straten, Irma M. Verdonck-de Leeuw, the NET-QUBIC consortium

**Affiliations:** 1 Department of Clinical, Neuro- and Developmental Psychology, Faculty of Behavioural and Movement Sciences & Amsterdam Public Health Research Institute, Vrije Universiteit Amsterdam, Van der Boechorststraat 7, 1081 BT Amsterdam, The Netherlands; 2 Cancer Center Amsterdam Research Institute, Amsterdam UMC, Vrije Universiteit Amsterdam, Amsterdam, The Netherlands; 3 Department of Otolaryngology - Head and Neck Surgery, Amsterdam UMC, Vrije Universiteit Amsterdam, PO Box 7057, 1007 MB Amsterdam, The Netherlands; 4 Department of Epidemiology and Biostatistics, Amsterdam UMC, Vrije Universiteit Amsterdam, Amsterdam, The Netherlands; 5 Department of Otolaryngology and Head and Neck Surgery, Erasmus Cancer Institute, Erasmus MC, Rotterdam, The Netherlands; 6 Department of Radiation Oncology, University of Groningen, University Medical Centre Groningen, Groningen, The Netherlands; 7 Department of Psychiatry, Amsterdam Public Health, Amsterdam UMC, Vrije Universiteit Amsterdam, Amsterdam, The Netherlands; 8 Department of Otorhinolaryngology and Head and Neck Surgery, Radboud Institute for Health Sciences, Radboud University Medical Center, Nijmegen, The Netherlands; 9 Department of Radiotherapy, University Medical Center, Utrecht, The Netherlands; 10 Department of Otolaryngology - Head and Neck Surgery, Project Kubus, Amsterdam UMC, Vrije Universiteit Amsterdam, Amsterdam, The Netherlands

**Keywords:** Sleep quality, Head and neck cancer, Newly diagnosed, Before treatment

## Abstract

**Background:**

Head and neck cancer (HNC) patients often suffer from distress attributed to their cancer diagnosis which may disturb their sleep. However, there is lack of research about poor sleep quality among newly diagnosed HNC patients. Therefore, our aim was to investigate the prevalence and the associated factors of poor sleep quality among HNC patients before starting treatment.

**Materials and methods:**

A cross-sectional study was conducted using the baseline data from NET-QUBIC study, an ongoing multi-center cohort of HNC patients in the Netherlands. Poor sleep quality was defined as a Pittsburgh Sleep Quality Index (PSQI) total score of > 5. Risk factors examined were sociodemographic factors (age, sex, education level, living situation), clinical characteristics (HNC subsite, tumor stage, comorbidity, performance status), lifestyle factors, coping styles, and HNC symptoms.

**Results:**

Among 560 HNC patients, 246 (44%) had poor sleep quality before start of treatment. Several factors were found to be significantly associated with poor sleep: younger age (odds ratio [OR] for each additional year 0.98, 95% CI 0.96–1.00), being female (OR 2.6, 95% CI 1.7–4.1), higher passive coping style (OR 1.18, 95% CI 1.09–1.28), more oral pain (OR 1.10, 95% CI 1.01–1.19), and less sexual interest and enjoyment (OR 1.13, 95% CI 1.06–1.20).

**Conclusion:**

Poor sleep quality is highly prevalent among HNC patients before start of treatment. Early evaluation and tailored intervention to improve sleep quality are necessary to prepare these patients for HNC treatment and its consequences.

## Introduction

More than 800,000 people worldwide were newly diagnosed with head and neck cancer (HNC) in 2018 [1]. There is a growing attention to maximize health-related quality of life (HRQoL) of newly diagnosed HNC patients [2]. These patients often suffer from emotional distress and concerns related to the future consequences of HNC itself and its treatment [3, 4], which may affect their sleep quality. Sleep quality before the start of cancer treatment is also known to be associated with HRQoL throughout the cancer trajectory [5, 6]; thus, early detection of poor sleep quality is necessary to initiate prehabilitation strategy to optimize HRQoL during and after HNC treatment [7].

Nonetheless, little is known about sleep quality among newly diagnosed HNC patients. A recent systematic review found a wide prevalence range of 16 to 66% for various definitions of sleep disturbances among HNC patients before treatment [8]. Additionally, most of the included studies did not use validated instrument to measure sleep quality as reported by the patients themselves [8]. To illustrate, only two studies among newly diagnosed HNC patients used the Pittsburgh sleep quality index (PSQI) [9, 10], a validated and most widely used self-report instrument for sleep quality in clinical and non-clinical populations [11]. Using a PSQI total score cut-off of > 5, these two studies found that 37% of their patients had poor sleep quality [9, 10]. Since these studies only included nasopharyngeal cancer patients [9, 10], the prevalence of poor sleep quality among a more generalizable sample of newly diagnosed HNC patients is yet to be examined.

Furthermore, more insight is needed on the factors associated with poor sleep quality among HNC patients before treatment. Only two studies have examined this question thus far and found that age, marital status, HNC subsite, smoking status, and physical activity were significantly associated factors [12, 13], implying the importance to assess these factors in sleep quality evaluation among newly diagnosed HNC patients. Neither of these studies, however, examined two important factors among newly diagnosed HNC patients: coping styles and HNC symptoms. Coping styles determine how someone perceives stressful life events, such as being diagnosed with cancer. Although the effectiveness of coping style may depend on the context of the stressor, certain coping styles such as avoidance coping, substance use, and behavioral and mental disengagement are found to be associated with more psychological distress among HNC patients before starting treatment [14]. Avoidance coping style in particular is found to be associated with poor sleep among cancer patients in general [15, 16]. So far, there is no research on whether specific coping styles are associated with poor sleep quality among newly diagnosed HNC patients. Furthermore, patients recently diagnosed with HNC often suffer from oral pain and swallowing problems [17] which may disrupt their sleep quality.

Insight into poor sleep quality among newly diagnosed HNC patients may help healthcare providers to design a better sleep intervention for those who already need it before starting treatment. Therefore, we aimed to examine the prevalence of poor sleep quality among HNC patients before start of treatment and to examine the association of poor sleep quality with sociodemographic factors, clinical characteristics, lifestyle factors, coping style, and HNC-specific symptoms.

## Patients and methods

### Study population

Data of the prospective NETherlands QUality of life and BIomedical Cohort study in head and neck cancer (NET-QUBIC) [18] was used. In the NET-QUBIC study, 739 newly diagnosed HNC patients from HNC centers in 5 university medical centers and 2 of their satellite hospitals in the Netherlands were included between March 2014 and June 2018. Inclusion criteria were as follows: being diagnosed with squamous cell carcinoma of the oral cavity, oropharynx, hypopharynx, larynx, or neck lymph node metastasis of an unknown primary tumor; being 18 years or older; having curative treatment intention; and being able to write, read, and speak Dutch. Exclusion criteria were having severe psychiatric comorbidity (e.g., schizophrenia, Korsakoff’s syndrome, severe dementia), lymphoma, thyroid cancer, nasopharyngeal cancer, malignancy of skin, or malignancy of salivary glands. The NET-QUBIC study was approved by the Medical Research Ethics Committee of the coordinating center (Amsterdam UMC, location VUmc, document number: 2013.301[A2018.307]-NL45051.029.13). Detailed procedure of the NET-QUBIC study can be found elsewhere [18].

### Measures

The ongoing NET-QUBIC study encompasses measurements at baseline and at 3-, 6-, 12-, 24-, 36-, 48-, and 60-month follow-up. In the present study, we used the baseline data, which were collected shortly after the diagnosis and before cancer treatment was started.

Sleep quality was measured using the PSQI, which was filled in by the patients themselves [19]. Its validity and reliability have been confirmed both in general population [20] and in cancer patients [21, 22]. PSQI consists of 19 items across seven components of sleep quality and disturbances: (1) subjective sleep quality (i.e., one item “How would you rate your sleep quality overall?”), (2) sleep onset latency (i.e., two items asking time needed to fall asleep and its frequency in a week; poor sleep onset is defined as needing ≥ 30 min to fall asleep [23]), (3) sleep duration (i.e., one item “How many hours of actual sleep do you get at night?”; ≤ 6 h is associated with worse survival among cancer patients [24]), (4) sleep efficiency (i.e., a percentage calculated by dividing time asleep by time spent in bed and multiplied by 100; < 85% indicates poor efficiency [23]), (5) sleep disturbances (i.e., ten items about specific reasons for the sleep disturbances and their frequency), (6) use of sleep medication (i.e., one item “How often have you taken medicine to help you sleep?”), and (7) daytime dysfunction (i.e., two items asking the frequency of staying awake during daytime activity and the extent of difficulty to maintain enthusiasm to get things done) [19]. A total score (also called global score) is calculated by first scaling each component score into a 0 to 3 score then summing all component scores, resulting in a score ranging from 0 to 21; higher scores indicate worse sleep quality. Our main outcome, poor sleep quality, is defined by a total PSQI score of > 5 [19, 20]. In addition, we examined questions in the PSQI (not included in the total score calculation) which assessed the frequency of the respondent’s bed partner/roommate noticing certain behaviors during the respondent’s sleep. This information is clinically relevant as an indication of specific sleep disorders such as sleep apnea [25].

We examined the following sociodemographic factors: sex and age (from medical records), living situation (living together/alone, from interview), education level (low/middle/high according to the standard classification of education level in the Netherlands [26], from interview), and having a bed partner (yes/no, from the PSQI). The interview was conducted by trained field workers during house visit measurements. This interview also included other outcomes which were out of the scope of the present study [18]. Clinical characteristics (i.e., HNC subsite and stage, comorbidity, and performance status) were retrieved from medical records. Comorbidity was scored using the adult comorbidity evaluation (ACE-27). The ACE-27 measures the number and severity of 27 medical conditions, and is summarized into four categories: no comorbidity, mild comorbidity, moderate comorbidity, or severe comorbidity [27]. The ACE-27 has been validated among HNC patients [28]. The patients’ performance status (i.e., the patient’s level of functioning seen from their daily activity, physical ability, and self-care) was measured using the one-item Eastern cooperative oncology group (ECOG) score, which ranges from 0 (fully active) to 4 (completely disabled) [29].

Coping styles were measured by the self-reported 47-item Utrecht coping list (UCL) [30]. Each item ranges from 0 (never or seldom) to 3 (very often). The UCL measures active coping (i.e., evaluating the situation from all perspectives and taking action to solve the problem; 7 items), palliative reaction (i.e., finding distractions against the problem and seeking for manners to feel better; 8 items), avoidance coping (i.e., avoiding the situation and letting it to be solved by itself; 8 items), seeking social support (i.e., seeking help and understanding from the others, expressing worries; 6 items), passive coping (i.e., taking the blame for the situation, worrying about things in the past, withdrawing into oneself; 7 items), expression of emotions (i.e., expressing anger or abreaction; 3 items), and comforting thoughts (i.e., assuring one’s self that things will get better, that things could have been worse, or that the others’ may also have similar difficulties; 5 items) [30, 31]. For each coping style, a sum score was calculated, where a higher score indicates higher extent the specific coping style.

HNC symptoms were self-reported using the European organization for research and treatment of cancer quality of life questionnaire - HNC-specific module (EORTC QLQ-H&N35) [32]. All symptom scales were included: oral pain (4 items), swallowing problems (4 items), sense problems (2 items), speech problems (3 items), trouble with social eating (4 items), trouble with social contact (5 items), and less sexual interest and enjoyment (2 items). Also, we included Likert-scale single items measuring teeth problems, problems with opening mouth, dry mouth, sticky saliva, coughing, and feeling ill, as well as dichotomous single items measuring use of painkillers, use of nutritional supplements, use of feeding tube, weight loss, and weight gain. Each symptom scale and Likert-scale single item was converted into a score ranging from 0 to 100, according to the EORTC guidelines. A higher score indicates a higher level of symptoms or problems.

The following lifestyle factors were examined: physical activity, body mass index (BMI), smoking status, and alcohol intake. Physical activity was assessed using the 13-item physical activity scale for the elderly (PASE) with total score ranging from 0 to 400; a higher score indicates higher physical activity [33]. The validity and reliability of this patient-reported outcome measure has been previously confirmed among cancer patients [34]. BMI was measured by a trained field worker using a standardized procedure during house visit. Smoking status (not smoking/smoking every day at the time of assessment) and excessive alcohol consumption (> 14 units of alcohol per week for women or > 21 units of alcohol per week for men [35]) were self-reported using study-specific items.

### Statistical analysis

Only the participants who completed the main outcome measure (PSQI) were included in the analysis. Sociodemographic and clinical characteristics of participants who were included versus excluded from the analysis were compared using *t* test (for continuous variables) or chi-square test (for categorical variables). For the included patients, we performed descriptive analyses: mean scores with standard deviation (SD) or medians with interquartile range (IQR) for continuous variables, and frequencies with proportions for categorical variables. We also examined the proportions and mean scores (with SD) or median (with IQR) of the PSQI total and component scores.

To examine the differences among patients with good and poor sleep, first, their sociodemographic, clinical, and lifestyle characteristics, as well as coping styles and HNC symptoms, were compared using chi-square (categorical variables) and *t* tests or Mann-Whitney tests (continuous variables). Then, forward multivariable logistic regression analyses were performed on each category of associated factors separately (i.e., sociodemographic, coping style, clinical factors, HNC symptoms, and lifestyle factors) using a *p* value for entry of < 0.05. Subsequently, we performed logistic regression analyses using a forward selection of all significant variables across all categories, resulting in the final model. In all regression models, HNC symptom and item scores which ranged from 0 to 100 were rescaled into 10-point increments. All statistical analysis was performed using the IBM SPSS Statistics for Windows, Version 25.0 (Armonk, NY: IBM Corp. 2017).

## Results

### Study population

Among the 739 eligible patients, 179 (24%) did not have available PSQI scores and were excluded from our analyses. Age, gender, education level, HNC subsite, and HNC stage were similar between patients with (*n* = 560, 76%) and without PSQI data. However, patients providing PSQI data more often lived with others, had better ECOG performance status, and less comorbidity compared with the patients not providing PSQI data (Table 1). There were 171 (21%) missing values on BMI; therefore, this variable was not included in the analysis. For all other variables, missing values ranged from 7 (1.3%) to 43 (7.7%). Since only 5 patients (0.9%) used a feeding tube, we did not include this variable in the analysis.

**Table 1:** Characteristics of patients with available versus unavailable PSQI data

	Available PSQI data (*n* = 560)	Unavailable PSQI data (*n* = 179)	*p* value
Age (mean, SD)	63 (9)	63 (11)	0.33
Women	142 (25%)	48 (27%)	0.70
Education level^a,b^			
Low	215 (42%)	64 (49%)	0.19
Middle	136 (26%)	35 (27%)	
High	166 (32%)	32 (24%)	
Living alone^a^	108 (21%)	56 (43%)	< 0.001
HNC location			
Oral cavity	157 (28%)	42 (24%)	0.34
Oropharynx^c^	198 (35%)	64 (36%)	
Hypopharynx	35 (6%)	17 (10%)	
Larynx	152 (27%)	53 (30%)	
Unknown primary	18 (3%)	3 (2%)	
HNC stage			
I	134 (24%)	29 (16%)	0.086
II	103 (18%)	29 (16%)	
III	90 (16%)	37 (21%)	
IV	233 (42%)	84 (47%)	
ECOG performance status			
0	398 (71%)	109 (61%)	0.012
1 or more	162 (29%)	70 (39%)	
Comorbidity^a^			
None	172 (32%)	32 (19%)	0.002
Mild	203 (38%)	61 (37%)	
Moderate	109 (20%)	46 (28%)	
Severe	50 (9%)	26 (16%)	

^a^There were 91 missing values on education level, 90 missing values on living arrangement, and 40 missing values on comorbidity

^b^Low education level includes primary education, lower or preparatory vocational education, and intermediary general secondary education. Middle education level includes senior general secondary education and higher general secondary education. High education level includes higher professional education and university

^c^Human papilloma virus (HPV) status of oropharynx cancer patients with available PSQI data was positive among 104 patients, negative among 67 patients, and not tested among 27 patients. For patients with unavailable PSQI data, the HPV status was positive among 26 patients, negative among 32 patients, and not tested among 6 patients

Our final sample of patients with PSQI data were mostly men (75%), on average 63 years old (SD = 9), and low educated (42%). Most patients (79%) lived with others (i.e., with a partner, child, and/or housemate) and 70% had a bed partner. About one-third (32%) had no comorbidity (Table 3).

### Sleep quality

The median PSQI total score was 5 (IQR = 3–8). There were 246 HNC patients who were classified as having poor sleep (PSQI > 5; 44%). The median time needed to fall asleep (i.e., sleep latency) was 13 min (IQR = 8–30) and the mean sleep duration was 7 h (SD = 1). Among all patients, 13% slept ≤ 6 h in a night, 42% had < 85% sleep efficiency, 16% could not fall asleep within 30 min at least three nights a week, and 45% reported nighttime or early morning awakening at least three times a week. At least once in a week, 15% of the patients used medication to improve their sleep and 5% experienced difficulties staying awake during the day. Questions answered by 417 bed partners or roommates revealed that 173 (43%) patients snored loudly and 13% of the patients had long breathing pause at least once a week. Mean (SD) or median (IQR), as well as the proportions of each component score, are presented in Table 2.

**Table 2:** Overview of PSQI components

PSQI component		Mean (SD) or median (IQR)	*n* (%)
Subjective sleep quality^a^		NA	
Component score	0 (very good)		137 (25)
	1 (fairly good)		303 (54)
	2 (fairly bad)		107 (19)
	3 (very bad)		12 (2)
Sleep latency, in minutes^b^		13 (8–30)	
Component score*	0		208 (39)
	1		196 (36)
	2		87 (16)
	3		47 (9)
Sleep duration, in hours^c^		7.1 (1.3)	
Component score	0 (> 7 h)		365 (65)
	1 (6–7 h)		122 (22)
	2 (5–6 h)		44 (8)
	3 (< 5 h)		26 (5)
Sleep efficiency^d^		NA	
Component score*	0 (> 85%)		322 (58)
	1 (75–84%)		125 (23)
	2 (65–74%)		42 (7)
	3 (< 65%)		64 (12)
Sleep disturbances^e^		NA	
Component score*	0		23 (4)
	1		328 (59)
	2		185 (33)
	3		22 (4)
Use of sleep medication^a^		NA	
Component score	0 (not during the past month)		460 (82)
	1 (< 1/week)		15 (3)
	2 (1–2/week)		28 (5)
	3 (≥ 3/week)		56 (10)
Daytime dysfunction^a^		NA	
Component score*	0		250 (45)
	1		256 (46)
	2		50 (9)
	3		3 (0.5)
Questions answered by bed partner/roommate about patients’ sleep behavior (*n* = 417) ^f^		NA	
	Loud snoring ≥ 1/week		173 (43)
	Long breathing pause ≥ 1/week		50 (12)
	Legs twitching ≥ 1/week		35 (9)
	Episodes of disorientation ≥ 1/week		4 (1)

*Component score is calculated from ≥ 2 items and rescaled to 0 to 3. Higher component score denotes worse complaints

^a^Subjective sleep quality, use of sleep medication, and day dysfunction due to sleepiness could not be calculated for 1 patient

^b^Sleep latency could not be calculated for 22 patients

^c^Sleep duration could not be calculated for 3 patients

^d^Sleep efficiency could not be calculated for 7 patients

^e^Sleep disturbances could not be calculated for 2 patients

^f^Missing from 11 patients on snoring, 16 on breathing pauses, 14 on legs twitching, and 12 on episodes of disorientation

*Abbreviations*: *PSQI*, Pittsburgh Sleep Quality Index; *NA*, not applicable; *SD*, standard deviation

### Factors associated with poor sleep quality

Univariate analyses revealed that patients with poor sleep (*n* = 246) were more often women, younger, diagnosed with cancer in the oral cavity, using painkillers, or using nutritional supplements (Table 3). Furthermore, compared with good sleepers, poor sleepers used less active coping and more palliative reaction, passive coping, and expression of emotions. Also, poor sleepers had worse scores of all HNC symptoms except for speech problems, problems with opening mouth, coughing, and weight changes.

**Table 3:** Characteristics and group comparisons of patients with good sleep versus patients with poor sleep

	All patients (*n* = 560)	Good sleep (*n* = 314)	Poor sleep (*n* = 246)	*p* value
Age (mean, SD)	63 (9)	64 (9)	62 (9)	0.020
Women, *n* (%)	142 (25)	51 (36)	91 (64)	0.001
Education, *n* (%)^a, b^				
Low	215 (42)	117 (54)	98 (46)	0.40
Middle	136 (26)	84 (62)	52 (38)	
High	166 (32)	95 (57)	71 (43)	
Living alone, *n* (%)^a^	108 (21)	59 (55)	49 (45)	0.59
Had bed partner, *n* (%)^a^	385 (70)	223 (58)	162 (42)	0.16
Coping, mean (SD) or median (IQR)^a^				
Active coping	11.7 (4)	12 (4)	11.3 (4)	0.035
Palliative reaction	9.3 (3)	9 (4)	9.8 (4)	0.006
Avoidance coping	7.3 (4)	7 (3)	7.5 (3)	0.068
Seeking social support	6.9 (3)	6.8 (3)	7.1 (3)	0.27
Passive coping	3 (1–5)	2 (1–4)	3 (2–6)	< 0.001
Expression of emotions	2 (1–3)	1 (0–3)	2 (1–3)	0.001
Comforting thoughts	7.3 (2)	7.1 (2)	7.5 (3)	0.078
HNC location, *n* (%)				
Oral cavity	157 (28)	74 (47)	83 (53)	0.008
Oropharynx	198 (35)	108 (55)	90 (45)	
Hypopharynx	35 (6)	19 (54)	16 (46)	
Larynx	152 (27)	103 (68)	49 (32)	
Unknown primary	18 (3)	10 (56)	8 (44)	
HNC stage, *n* (%)				
I	134 (24)	84 (63)	50 (37)	0.24
II	103 (18)	59 (57)	44 (43)	
III	90 (16)	45 (50)	45 (50)	
IV	233 (42)	126 (54)	107 (46)	
ECOG performance status, *n* (%)				
0	398 (71)	231 (58)	167 (42)	0.16
1 or more	162 (29)	83 (51)	79 (49)	
Comorbidity, *n* (%)^a^				
None	172 (32)	102 (59)	70 (41)	0.42
Mild	203 (38)	113 (56)	90 (44)	
Moderate	109 (20)	62 (57)	47 (43)	
Severe	50 (9)	23 (46)	27 (54)	
Head-neck symptoms, mean (SD) or *n* (%)^a^				
Oral pain	26 (24)	22 (22)	32 (26)	< 0.001
Swallowing problems	16 (21)	13 (20)	19 (22)	< 0.001
Sense problems	8 (17)	7 (14)	10 (19)	0.008
Speech problems	19 (23)	17 (22)	20 (24)	0.16
Problems with social eating	11 (17)	8 (15)	15 (19)	< 0.001
Problems with social contact	4 (10)	3 (7)	6 (12)	< 0.001
Less sexual interest and enjoyment	26 (31)	20 (27)	34 (34)	< 0.001
Teeth problems	16 (27)	13 (25)	19 (30)	0.013
Problems with opening mouth	12 (25)	10 (22)	14 (28)	0.053
Dry mouth	16 (23)	13 (21)	19 (25)	0.001
Sticky saliva	14 (24)	12 (22)	17 (25)	0.017
Coughing	23 (24)	22 (22)	25 (26)	0.11
Feeling ill	13 (23)	9 (18)	18 (24)	< 0.001
Used painkillers^c^	299 (54%)	141 (47%)	158 (53%)	< 0.001
Used nutritional supplements^c^	86 (16%)	36 (42%)	50 (58%)	0.006
Had weight loss^c^	133 (24%)	65 (49%)	68 (51%)	0.056
Had weight gain^c^	57 (10%)	29 (51%)	28 (49%)	0.40
Smoking daily, *n* (%)^a^	123 (22)	62 (50)	61 (50)	0.18
Excessive alcohol consumption, *n* (%)^a^	124 (22)	73 (59)	51 (41)	0.54
PASE global score, median (IQR)^a^	84 (43–144)	83 (46–134)	90 (39–154)	0.51

^a^Variables with missing values: 43 missing values on education, 42 on living arrangements, 7 on having bed partner, 4–10 on each UCL domain scores, 26 on comorbidity, 7 on all EORTC-H&N35 domains (except less sexual interest and enjoyment [missing = 43], teeth problems [missing = 12], problems with opening mouth [missing = 9], sticky saliva [missing = 8], coughing [missing = 8], using painkillers [missing = 10], using feeding tube [missing = 8], had weight loss [missing = 11], and had weight gain [missing = 14]), 9 on smoking status, 7 on alcohol consumption, 7 on PASE score, 11 on snoring, and 16 on breathing pauses

^b^Low education level includes primary education, lower or preparatory vocational education, and intermediary general secondary education. Middle education level includes senior general secondary education and higher general secondary education. High education level includes higher professional education and university

^c^Item with yes/no answer response; the proportions of “yes” were reported in the table

*Abbreviations*: *HNC*, head and neck cancer; *PASE*, physical activity scale for the elderly; *SD*, standard deviation; *UCL*, Utrecht Coping List

Multivariate logistic regression models on the separate domains of risk factors showed that poor sleep was significantly associated with sociodemographic factors (younger age, being female), coping style (more passive coping), clinical characteristics (HNC subsite, especially cancer in the oral cavity and oropharynx compared with larynx), and HNC symptoms (oral pain, less sexual interest and enjoyment, and feeling ill). Combining all of these significant variables in a logistic regression model, we found that younger age (odds ratio [OR] per increasing year of age = 0.98, 95% CI = 0.96−1.00, *p* value = 0.049), being female (OR = 2.6, 95% CI = 1.7–4.1, *p* value < 0.001), higher passive coping style (OR = 1.18, 95% CI = 1.09–1.28, *p* value < 0.001), more oral pain (OR = 1.10, 95% CI = 1.01–1.19, *p* value = 0.023), and less sexual interest and enjoyment (OR = 1.13, 95% CI 1.06–1.20, *p* value < 0.001) corresponded to greater odds of having poor sleep (Table 4).

**Table 4:** Final model of the multivariable logistic regression analyses with poor sleep (PSQI total score> 5) as outcome (*n* = 516)

Covariates^a^	OR	*p* value	95% CI
Age	0.98	0.049	0.96–1.00
Female	2.6	< 0.001	1.7–4.1
Passive coping	1.18	< 0.001	1.09–1.28
Oral pain^b^	1.10	0.023	1.01–1.19
Less sexual interest and enjoyment^b^	1.13	< 0.001	1.06 – 1.20

^a^Covariates retained in the final model is based on forward selection of all significant variables of each category, with entry criteria *p* value of < 0.05. Nagelkerke’s *R* = 0.21

^b^Oral pain and less sexual interest and enjoyment scores were transformed into 10-point increments

*Abbreviations*: *CI*, confidence interval; *OR*, odds ratio; *PSQI*, Pittsburgh Sleep Quality Index

## Discussion

In this study, we examined the prevalence of poor sleep quality among HNC patients before start of treatment and investigated the associated factors. Almost half of the patients in this study were categorized as poor sleepers. This prevalence is higher than prevalence in the general population (36%) [20], thus emphasizing the importance to incorporate sleep evaluation shortly after HNC diagnosis. Regarding the PSQI component scores, we found that a high proportion of patients reported poor sleep efficiency, difficulty to fall asleep, and nighttime or early morning awakenings; these three complaints are particularly relevant as they are the most commonly reported symptoms of insomnia [23]. Comparing our prevalence rates with those of similar studies was not possible because no other study has been published using both a similar population of HNC patients and a validated self-reported measure for sleep quality.

We found that younger age, being female, having passive coping style, more oral pain, and less sexual interest and enjoyment were the most significant factors associated with poor sleep. In the general population, older adults tend to experience age-related changes in sleep-wake architecture, such as more sleep awakenings, less deep sleep, and less sleep efficiency [23]. This is apparently not the case for HNC patients, as we found that it was younger patients who had worse sleep quality. This is in line with findings from two previous studies among HNC patients before treatment [12, 13]. These findings may be attributed to fear of cancer progression which is higher among younger HNC patients before treatment [36]. Patients who are diagnosed with cancer at a younger age also experience higher psychological distress compared with their older counterparts [37], which may contribute to their higher risk of having poor sleep.

The greater odds of having poor sleep among women in our study is consistent with findings among general population in the Netherlands [38]. Differences in the physiology of sex hormones as well as circadian rhythms among men and women may explain the differences of sleep quality between the sexes [39]. However, previous studies examining poor sleep among pre-treated HNC patients reported different findings. The cause of this disagreement is unclear, although it may be in part due to the differences in defining sleep quality and the associated factors examined in the study. Using a single item (“How much is a problem is sleeping for you?”), Zhou and colleagues compared sex proportions of patients with severe sleep problem (*n* = 45) with those who had no, mild, or moderate problems (*n* = 281); they found that slightly more females had severe sleep problems, although this was not statistically significant [13]. These findings suggest that a dichotomized Likert-scale item is not informative enough to illustrate sex differences in poor sleep quality. On the other hand, Duffy and colleagues, who also looked at depressive symptoms in relation to sleep quality, found that depressive symptoms, and not sex, are associated with sleep quality [12]. Whether sex differences on sleep quality are completely attributed to depressive symptoms remains questionable, since depressive symptoms among both men and women seem to affect sleep quality in different ways [40, 41]. More research is needed to assess the complex relationship of depressive symptoms and sleep quality among men and women with HNC.

We found a significant association between poor sleep and passive coping style. Diagnosis of cancer, as a form of distress, may be perceived differently among HNC patients and its effect on sleep quality may be determined by coping style. No specific coping style is inherently positive or negative since certain coping styles can be effective respective of the onset and context of the distress [42]. However, coping strategies such as avoidance, denial, substance use, abreaction, and behavioral and mental disengagement are known to be related to psychological distress among newly diagnosed HNC patients [14, 43]. We found that sleep quality before HNC treatment is associated with passive coping style, which comprises worrying about things in the past, withdrawing into oneself, and taking the blame for the situation. Passive coping style is also found to be associated with psychological distress after HNC treatment [44]. Although our cross-sectional study may not be able to explain any causal relationship, a longitudinal research among general population found that substance use, behavioral disengagement, and distracting oneself, compared with other coping styles, are associated with higher risk of having insomnia after stress exposure [45]. Whether this is also the case for HNC patients after treatment starts remains to be examined in future research.

As expected, based on clinical practice, we found significant associations between poor sleep and HNC symptoms, more specifically oral pain and less sexual enjoyment. The relationship between pain, sexual problems, and sleep problems may be bidirectional. First of all, pain may disrupt sleep, while poor sleep may also lower one’s pain threshold, thus increasing the perception of pain [46]. Sexual satisfaction may improve sleep quality through the release of oxytocin and prolactin levels, both of which are neuropeptides involved in sleep regulation [47]. Lack of sleep, on the other hand, may result in fatigue and reduce sexual desire [48]. More research is needed to confirm whether sleep intervention before treatment can optimize symptom management in HNC patients throughout the cancer trajectory.

### Strength and limitations

The strength of this study was that we used a large sample of 560 HNC patients from multiple hospitals in the Netherlands to investigate the association between sleep problems and various sociodemographic, clinical, and lifestyle factors as well as coping style and HNC symptoms. Moreover, our study was the first study so far to report on the prevalence of different components of sleep quality (as measured by the PSQI) among HNC patients before starting treatment. However, we found differences between patients who were included because their PSQI data were available (*n* = 560) compared with those that did not (*n* = 179); our study population consists of HNC patients with better performance status and less severe comorbidity. Our study may, therefore, underestimate the real prevalence of poor sleep quality among newly diagnosed HNC patients. In addition, the NET-QUBIC participants were not completely representative for the overall HNC population in the Netherlands [18], which may limit generalizability of our study findings to all HNC patients.

## Conclusions

Poor sleep quality is highly prevalent among newly diagnosed HNC patients and is associated with younger age, being female, passive coping style, more oral pain, and less sexual interest and enjoyment. Our findings underline the need for early sleep evaluation among HNC patients already before starting treatment, as well as taking coping styles and HNC symptoms into consideration when implementing sleep intervention.

## Abbreviations

ACE-27: Adult comorbidity evaluation
BMI: Body mass index
ECOG: Eastern cooperative oncology group
EORTC QLQ-H&N35: European organization for research and treatment of cancer quality of life questionnaire - HNC-specific module
HNC: Head and neck cancer
HRQoL: Health-related quality of life
IQR: Interquartile range
NET-QUBIC: The NETherlands QUality of life and BIomedical Cohort study in head and neck cancer
PASE: Physical activity scale for the elderly
PSQI: Pittsburgh sleep quality index
SD: Standard deviation
UCL: Utrecht coping list
